# Managing the effects of multiple stressors on wildlife populations in their ecosystems: developing a cumulative risk approach

**DOI:** 10.1098/rspb.2022.2058

**Published:** 2022-11-30

**Authors:** Peter L. Tyack, Len Thomas, Daniel P. Costa, Ailsa J. Hall, Catriona M. Harris, John Harwood, Scott D. Kraus, Patrick J. O. Miller, Michael Moore, Theoni Photopoulou, Enrico Pirotta, Rosalind M. Rolland, Lori H. Schwacke, Samantha E. Simmons, Brandon L. Southall

**Affiliations:** ^1^ Sea Mammal Research Unit, School of Biology, Scottish Oceans Institute, University of St Andrews, St Andrews, UK; ^2^ Centre for Research into Ecological and Environmental Modelling, University of St Andrews, St Andrews, UK; ^3^ SMRU Consulting, Scottish Oceans Institute, University of St Andrews, St Andrews, UK; ^4^ Department of Ecology and Evolutionary Biology, University of California, Santa Cruz, CA, USA; ^5^ Institute of Marine Sciences, University of California, Santa Cruz, CA, USA; ^6^ Anderson-Cabot Center for Ocean Life, New England Aquarium, Boston, MA, USA; ^7^ Biology Department, Woods Hole Oceanographic Institution, Woods Hole, MA, USA; ^8^ Marine Mammal Commission, Bethesda, MD, USA; ^9^ Southall Environmental Associates, Inc., Aptos, CA, USA

**Keywords:** cumulative effects, cumulative risk, stressors, population, ecosystem, endangered species

## Abstract

Assessing cumulative effects of human activities on ecosystems is required by many jurisdictions, but current science cannot meet regulatory demands. Regulations define them as effect(s) of one human action combined with other actions. Here we argue for an approach that evaluates the cumulative risk of multiple stressors for protected wildlife populations within their ecosystems. Monitoring effects of each stressor is necessary but not sufficient to estimate how multiple stressors interact to affect wildlife populations. Examining the mechanistic pathways, from cellular to ecological, by which stressors affect individuals can help prioritize stressors and interpret how they interact. Our approach uses health indicators to accumulate the effects of stressors on individuals and to estimate changes in vital rates, driving population status. We advocate using methods well-established in human health and integrating them into ecosystem-based management to protect the health of commercially and culturally important wildlife populations and to protect against risk of extinction for threatened species. Our approach will improve abilities to conserve and manage ecosystems but will also demand significant increases in research and monitoring effort. We advocate for increased investment proportional to the economic scale of human activities in the Anthropocene and their pervasive effects on ecology and biodiversity.

## 1. Introduction

Estimating the effects of multiple stressors on wildlife is a vexing scientific problem of growing importance as changes to ecosystems are driven by increasing human impacts on the environment. The importance of cumulative effects is highlighted by a survey of more than 2000 scientists that identified cumulative effects of multiple stressors as the top global ocean research priority [1]. As the human population has grown, with expanding industrialization and agriculture, human activities are affecting terrestrial and marine ecosystems via multiple factors such as climate change, reduction of habitat, pollution and accidental injury or mortality, as well as directed hunting and fishing [2]. Managing the different kinds of potentially overlapping effects from these diverse activities requires methods to estimate their cumulative impact. This topic is also important for policy, environmental management and regulators. Environmental legislation since the 1970s has created legal requirements to assess cumulative effects. Consideration of cumulative effects is required for planners of major activities under Environmental Impact Assessment (EIA) and Environmental Impact Statement (EIS) processes of the European Union (EU), United States (US), United Kingdom (UK) and Canada [3], and is also important for agencies that develop recovery plans for threatened or endangered species, assess impacts of chemical spills (e.g. Ecological Risk Assessments), or develop restoration plans as part of natural resource damage assessments.

This paper aims to explain why different approaches exist to understand how these impacts accumulate over space and time, to clarify definitions, and to advocate an approach to managing cumulative effects. Our approach estimates the cumulative risk of multiple stressors on individuals within a wildlife population that is affected by ecological interactions with other species. Identifying how stressors contribute to cumulative risk can narrow the problem of estimating cumulative effects [4]. The ultimate goal of our approach is to identify management strategies that can rapidly and accurately decide when populations are vulnerable and identify combinations of stressors whose reduction can bring a threatened population to within an acceptable level of risk. Effective management requires the ability to identify when mitigation is required, which stressors to reduce and by how much. On the one hand, mitigation can have significant economic impacts and on the other hand, taking the wrong actions or implementing insufficient stressor reductions will fail to protect the managed population. Getting the balance wrong can either risk extinction of threatened species and damage to ecosystems or can create excess costs for human activities. Effects that propagate through the ecosystem can increase and spread these costs more broadly in our societies [5].

We argue that neither scientific theory nor data are up to the task, leading to failures of management that impose great costs on ecosystems and our societies. Significant new effort will be required to build the scientific basis for the approach we advocate. Therefore, we describe our approach not as something that can be implemented immediately, but rather as a call to build understanding and support for an ambitious and forward-looking framework designed to answer questions that are required by law and to guide future research that can improve management decisions that have major economic and ecological consequences. Our intended audience includes policy makers and managers of cumulative effects, as well as researchers from the diverse disciplines that assess risk and study effects of multiple stressors.

## 2. Differing regulatory definitions and their applications: cumulative effects of actions versus cumulative risk of stressors

**Figure 1.**
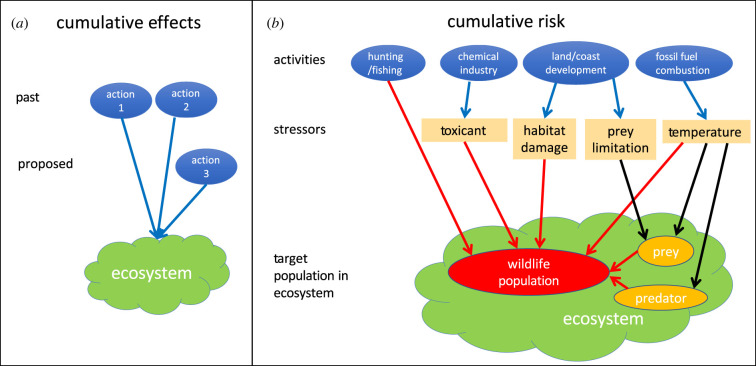
Illustration of differing definitions of cumulative effects and cumulative risk. (*a*) The action-based approach defines the cumulative effect of an action as the impact on the environment resulting from the incremental impact of the action when added to other past, present and reasonably foreseeable future actions. The arrows indicate cumulative effects of various actions taken by humans on an ecosystem, with effects of a new action 3 being added to the effects of existing actions 1 and 2. (*b*) The cumulative risk approach focuses on a wildlife population at risk from stressors. Human activities listed on the top row in blue produce stressors on the second row for a target population indicated by the red oval within its ecosystem indicated in green. The target population is also affected by biotic stressors such as predators and prey, indicated by the orange ovals within the ecosystem. When a stressor affects the wildlife population through another species, these indirect effects are indicated by a black arrow; direct effects are indicated by red arrows. (Online version in colour.)

Difficulties in evaluating cumulative effects are amplified by differing definitions of this and related terms among different communities. Regulations developed by the US Council on Environmental Quality (CEQ) define the *cumulative effect* of an action as ‘the impact on the environment which results from the incremental impact of the action when added to other past, present, and reasonably foreseeable future actions' ([6, p. 3]; note that phrases indicated in italic font are defined in box 1). The European Communities [10]) guide on cumulative effects and the Canadian Environmental Assessment Act of 1995 provide similar definitions. These definitions derive from the structure of EIAs, which analyse the effect of a proposed action and require proposers to analyse cumulative effects in the context of other pre-existing and planned actions (figure 1*a*).

Box 1. **Definitions used in this paper of terms that have varying usage by different communities that assess cumulative effects or cumulative risk. Sources for published definitions are given in parentheses.**

— **Action** – a human activity that creates stressors. Human actions and activities are typically what is regulated to maintain an environmental goal
— **Aggregate exposure** – the combined exposure of an individual (or defined population) to a specific agent or stressor via relevant routes, pathways and sources [7, p. 7]
— **Cumulative effect** – the impact on the environment which results from the incremental impact of an action when added to other past, present, and reasonably foreseeable future actions [6]
— **Cumulative risk** – the combined risk from aggregate exposures to multiple stressors
— **Dose** – the magnitude or amount of a stressor that is directly experienced, ingested, inhaled, or absorbed by an animal, ideally sampled in the animal or measured by a dosimeter on the animal [8, p. 133]
— **Dose–response function** – the relationship between the probability or severity of the response by a sample of individuals in a population depending on the dose they experienced
— **Effect** – when one or more responses to one or more stressors leads to a change in health condition or vital rates
  — **Immediate effect** – the effect of a stressor on an individual that occurs rapidly and is of short duration (called ‘acute effect’ in [8, p. 133])
  — **Protracted effect** – a stressor effect that does not immediately result in death or reproductive failure, but persists or is irreversible, and may influence long-term survival or reproductive success (called ‘chronic effect’ in [8, p. 133])
  — **Direct effect** – when considering the influences and interactions among species, and between species and their abiotic environment, direct effects are the proximate impacts that one species or factor has on another species or factor without the effect occurring via an intervening species or stressor [8, p. 133]
  — **Indirect effect** – an interaction between species or between species and the abiotic environment that occurs through one or more intervening species or abiotic factors [8, p. 134]
— **Exposure** – contact with or experience of a stressor, ideally measured in the environment near the animal
— **Health** – the ability of an organism to adapt to and manage threats to survival and reproduction
— **Interaction** – when the effect of one stressor is altered by another stressor
— **Population health** – the distribution of health outcomes in a population or subset of a population, as well as the determinants or factors that influence those outcomes
— **Response** – physiological or behavioural change made by an individual animal because of the dose of a stressor – can lead to an effect
— **Risk** – the probability of harmful effects to the health of individuals or to populations integrated over a defined time period
— **Stressor** – any physical, chemical or biotic entity that moves a biological system out of its normal operating range. stressors are often created by human actions and activities, and these may require regulation if environmental goals are to be attained or maintained (elements from [7] and [9]).

EIAs are prospective assessments that attempt to predict the future effects of a proposed action. In many nations, legislation to protect species at risk of extinction mandates prospective management plans for the recovery of endangered species. Other regulations may require retrospective or real-time analyses of cumulative effects. If a protected wildlife population has declined, regulations may mandate retrospective assessments to identify the cause(s) contributing to the decline. If a contaminated site has been releasing toxins for decades, retrospective analysis of the damage to the health of wildlife and to ecosystems may be required to determine how to remediate the damage and restore the ecosystem. Responses to chemical spills in the US often involve natural resource damage assessments that estimate present and future effects to determine the amount and type of restoration needed. Even in cases where the injury was caused by one action, such as overhunting or an oil spill, if the effects cannot be completely reversed by modifying that action alone, then restoration efforts may require reducing other stressors on the affected populations. Reducing alternative stressors requires prospective assessment of the potential benefit of reducing each alone or in combination. The importance of correctly modelling the benefits of reducing a set of stressors is highlighted by the tens of billions of dollars devoted to these restoration efforts in the US alone (e.g. [11]).

The problems and regulatory contexts described in the last paragraph are not well suited to the action-based approach for cumulative effects as defined in box 1 and figure 1*a*. Instead, they are like those faced by toxicologists assessing the risk from exposure to pollutants. When the US Environmental Protection Agency (EPA) assesses the risk to human health from exposure to chemicals that were released into the environment, this is called a chemical-focused assessment [7]. By contrast, when concern is raised about health effects in a human community, epidemiologists and toxicologists may perform retrospective analyses of what combinations of factors, including non-chemical factors, might have caused the health effects of concern [12]. The broadening of human health risk assessments to include non-chemical factors led EPA [7, p. 2] to adopt the term *stressor* to include ‘any physical, chemical, or biological entity that can induce an adverse response’. We take a broader definition of stressor in box 1; in our view, limitation of prey can be considered a stressor, even though prey does not induce an adverse response.

When assessing one stressor, EPA defines *risk* as ‘the chance of harmful effects to human health or to ecological systems resulting from exposure to an environmental stressor’ (see https://www.epa.gov/risk/about-risk-assessment#whatisrisk). Estimating the full measure of exposure to a stressor requires accounting for all potential sources and routes. EPA [7, p. 7] defines *aggregate exposure* as ‘the combined exposure of an individual (or defined population) to a specific agent or stressor via relevant routes, pathways and sources.’ The definition for *cumulative risk* commonly applied for risk assessment requires exposure to more than one stressor: ‘the combined risks from aggregate exposures to multiple agents or stressors' [7, p. 6].

## 3. Extending assessments of risk from individual stressors on human health to cumulative risk of multiple stressors on wildlife populations and ecosystems

Laws passed from the 1970s onwards have played an important role in broadening cumulative risk assessments from human health to include ecological applications. Regulatory demands to protect aquatic ecosystems led toxicologists to estimate the risk to aquatic organisms based upon their exposure to toxicants in the environment [13]. Legislation giving government agencies the authority to clean up sites contaminated by hazardous waste created demand to assess risk to species in the sites, which drove the development of ecological risk assessments [14]. Many of these sites and ecosystems were contaminated by more than one stressor. By the 1990s, regulatory demands led the EPA to broaden their cumulative risk assessments from impacts on human health to effects of a broad range of stressors on health of individuals, populations and ecosystems [7].

While the EPA was broadening cumulative risk assessments to whole ecosystems in the US, agencies in several countries responsible for managing cumulative effects worked on ways to integrate management of individual activities, stressors and effects together at the ecosystem level [15,16]. Ecosystem-based management developed in recognition of the weaknesses and inefficiencies of evaluating individual actions, stressors, or effects on a one-by-one basis. Most ecosystems today have been affected by so many activities for such a long time, that it is very difficult for the proponent of one action to meaningfully account for the cumulative effects of that action along with all the other effects of centuries of activities such as fishing, pollution, coastal and offshore development and global climate change.

Effective management of actions, stressors and effects requires understanding linkages between human activities, biophysical stressors and species. Figure 1*b* illustrates the connections between human activities in blue on the top row, with stressors in the middle row on a target wildlife population and other species they interact with in the ecosystem. This simple example shows how activity-based assessments must identify the stressors they produce and estimate effects they cause, whereas stressor-based assessments must identify the activities that produce them and the effects they cause. If an effect-based assessment aims to identify activities to change or actions to take to mitigate the effect, then it must also study the links between activities, stressors and effects.

Recognition of the need for regional assessments has led to the development of Integrated Ecological Assessments [16] and Cumulative Effect Assessments [17] in many jurisdictions. These typically start with a process to define the scope of the ecosystem (including human activities) to be managed, and critical targets and goals for management. This process generates a conceptual model that includes human activities that generate stressors which affect the ecosystem, as well as the species that are identified as high priority either because they are a target themselves or are ecologically linked to a target. Figure 1*b* illustrates a simple example of a conceptual model of how activities generate stressors that affect components of the ecosystem which affect goals for a managed population or ecosystem. The next step involves the selection of indicators that can be monitored to assess ecosystem status and to assess the cumulative risk that changes in stressors pose to key human and biological components of the ecosystem. If the cumulative effects of stressors are judged to pose too high a risk to management goals, management strategies are developed. Once a management action is taken, ecosystem indicators are monitored to test whether the action has the desired effect. By including human activities (both negative consequences of actions and positive consequences of mitigation), the conceptual model integrates humans as part of the ecological system [16,18].

## 4. Bibliometric analysis of cumulative effects and cumulative risk approaches to studying effects of multiple stressors

The broadening of the cumulative risk approach from human health to wildlife populations and ecosystems suggests that it may be relevant for studying cumulative effects. In this section, we explore the extent to which ecological assessments of cumulative effects have taken advantage of tools developed to study cumulative risk. Here, we use bibliometric analysis to examine the existing connections between research on these topics. To test the connections between research communities that approach the problems of multiple stressors in terms of cumulative effects and/or cumulative risk, we searched the Web of Science Core Collection for the terms ‘cumulative effects’ or ‘cumulative risk’ or ‘multiple stressors’ in the title or abstract of articles from 1980 to 2021. A total of 10 771 papers resulted and we selected the 1000 most cited papers for input into VOSviewer 1.6.16 (www.vosviewer.com). Details of how VOSviewer analyses relationships between terms are given in the electronic supplementary material.

**Figure 2.**
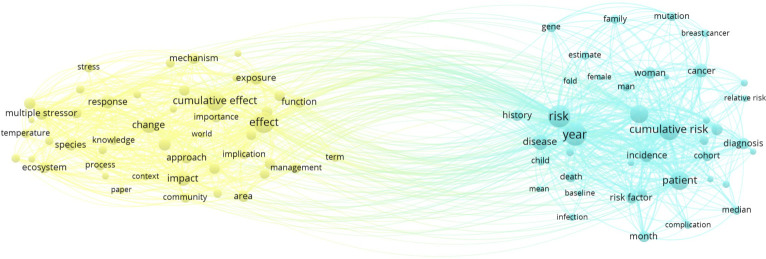
Network map of a bibliometric analysis of scientific papers extracted from a Web of Science search using the phrases ‘cumulative effects’, ‘cumulative risk’ or ‘multiple stressor(s)’. The terms illustrated here are noun phrases extracted from the titles and abstracts of each paper. Any plural forms were converted into singular to pool them into one term. The distance between a pair of terms is derived from the number of papers that share them with other papers. See the electronic supplementary material for more detail on bibliometric analyses. (Online version in colour.)

The network visualization shown in figure 2 illustrates the profound separation between the cumulative risk approach, whose cluster on the right is dominated by medical studies, and the cumulative effect approach, which clusters around environmental studies. In addition to the search for terms in titles and abstracts for papers, we also analysed the Web of Science categories for each paper. Papers associated with the search above for ‘cumulative risk’ show 4510 links to biomedical categories and only 842 links to environmental categories (lists of these categories and tables tallying the number of papers linked to categories associated with biomedical or environmental disciplines are in the electronic supplementary material). By contrast, results from the search for ‘cumulative effect’ or ‘cumulative effects’ yields 2434 links to biomedical categories and 3155 links to environmental categories. This suggests a weaker use of cumulative risk among environmental scientists than analysis of cumulative effects in medicine. Searches for ‘multiple stressors’ yield a greater gap between use for medicine with only 589 links and for environmental sciences with 2869 links. Our main point is that the powerful methods used by biomedical fields to evaluate cumulative risk are under-represented in research on cumulative effects of multiple stressors on wildlife and ecosystems. In fact, only 68 of the 8002 papers that cited either ‘cumulative effect(s)’ or ‘cumulative risk’ cited both.

Figure 2 shows that terms associated with the ‘cumulative effect’ cluster included ‘ecosystem’, ‘species’, ‘community’ ‘area’ and ‘environment’, suggesting an environmental focus, while those associated with ‘cumulative risk’ included ‘patient’, ‘woman’ and ‘man’, suggesting a medical focus. Examples of other terms associated with the ‘cumulative risk’ cluster were ‘year’, ‘risk’ and ‘age’, suggesting a focus on quantitative assessments of the probability that a patient would develop a disease over a defined time period as a function of risk factors such as age. By contrast, many of the terms associated with ‘cumulative effect’—‘approach’, ‘impact’, ‘change’, ‘management’ and ‘effect’—tend to be abstract and qualitative, related to policy issues.

This bibliographic analysis demonstrates that the terms ‘cumulative risk’ and ‘cumulative effect’ emerged from different communities. The different approaches and terms used by these communities pose an obstacle to adoption of methods across disciplines. Orr *et al*. [19] show how differences in terminology related to multiple stressors across different research disciplines have hindered scientific progress. Applying the science of multiple stressors to policy is made even more difficult by the different terms used to regulate cumulative effects and to assess cumulative risks. Here we aim to provide definitions that can be shared across communities, especially between policy makers and researchers (box 1).

## 5. Problems with current assessments of cumulative effects

Most wildlife managers are primarily concerned with the status and trends of populations. This requires understanding of how the various effects of multiple stressors combine to affect survival and reproduction, as well as how these translate to population dynamics. However, few assessments of cumulative effects actually attempt to estimate how different stressors will interact to affect individuals or populations. Studies reviewing how cumulative effects are addressed in EISs in the US [6] and UK [20,21] concluded that only a minority of EISs address cumulative effects, and when they do, it is only in qualitative terms. Only 16% of the cumulative effect assessments evaluated by Hague *et al*. [21] discussed *interactions* between stressors, and none of these actually estimated the cumulative risk from the combined stressors. EISs usually describe cumulative effects as additive, where the combined effect is equal to the added effect of each stressor in isolation; synergistic, where the combined effect is greater; or antagonistic, where the combined effect is less than the added individual effects. These distinctions stem from laboratory experiments that observe the effects of adding no stressor (control), one stressor A, another stressor B, or A and B combined. Meta-analyses of these experiments report few patterns that would help describe when interactions are additive or not [8]. Pirotta *et al*. [22] point out that these experiments seldom include more than one dose of each stressor, and unless the dose–response function is linear, the combined response may be less than, equal to, or greater than the sum of the independent responses simply depending upon what dosage is chosen for the experiment. These experiments are inadequate for estimating the combined effect of two or more stressors except under the specific dosages and conditions used. Estimation of cumulative effects to guide management decisions would require assessing the combined effects of the ranges of doses of stressors expected in the environment, including changes in doses recommended as mitigation measures. The problem for the action-based approach of attempting to predict the cumulative effects of introducing a new action that creates a set of stressors, on top of all existing actions and their associated stressors, is even less tractable than the problem of estimating the impact of two stressors presented together. The problem of estimating how multiple stressors interact to produce one combined effect is dwarfed by problems of interpreting how different effects from multiple stressors may interact to affect a wildlife population [19]. For example, reduced availability of prey may slow the growth and reproduction of a population while toxicants might increase mortality and decrease reproduction even more [23]. Some of these are *direct effects* of a stressor on the managed population; others are *indirect effects* that involve other species as well. Estimating and managing all these effects on a wildlife population requires an ecosystem-based analysis of cumulative risk.

## 6. Managing the cumulative risk to wildlife populations within their ecosystems

We propose an expanded role for incorporating assessments of cumulative risk to wildlife from multiple stressors into ecosystem-based management. We argue that managers of wildlife populations would benefit from quantitative approaches to cumulative risk similar to those used to protect human health (as identified in our bibliometric analysis, [24] and [7]). Using studies of cumulative risk to human health as a model, we argue that significant investment in strengthening theory and empirical research linking stressors to health and to population effects could greatly improve our ability to manage the risks that human activities have created for ecosystems. Here we propose an ambitious forward-looking approach that will require these investments. The models and data required by our expanded framework to improve decision-making are not yet available for many important applications. Management decisions need to be made every day, and we are not arguing for delay until perfect information is available. Even with a much larger scientific base, biomedical decision-makers must balance the cost of delay against the risks of approving suboptimal treatments. However, our health is greatly improved by higher demands for evidence of safety and effectiveness for treatments along with rejection of inaction, or blanket and costly remediation after the fact, as a default alternative. We urge readers concerned about ecological problems for their own applications to consider the cost of failing to clearly define and identify the consequences of stressor interactions and make appropriate decisions, either by demanding more costly solutions than are needed or by failing to protect the resource owing to inaction or implementation of ineffective measures. Then reconsider whether the costs of failure justify increased investment in better support for decision making.

Improving management of wildlife populations threatened by multiple stressors requires adapting methods from many scientific and policy-oriented disciplines. Box 2 lists a series of steps that we recommend for assessing cumulative risk to wildlife populations. These assessments are conducted to meet specific management goals deriving from regulations discussed above. Once management goals are defined, a scoping process defines a conceptual model that bounds the problem and makes assumptions explicit. Estimating the exposure of target population(s) to each stressor and estimating adverse effects can help to prioritize stressors of highest concern [25]. Stressors that have immediate effects on survival (e.g. hunting) or reproduction may be managed with monitoring of the stressors, the population and the ecosystem of concern. We propose an expanded framework for stressors whose effects may accumulate over time or whose interactions may be significant for management. In addition to the monitoring used in ecosystem-based management, we identify situations where management decisions will be improved by experiments designed to understand the mechanisms by which these stressors cause effects and to quantify dose–response functions. We advocate using health indicators to accumulate effects of these stressors. Analogous to methods used to protect human health, this framework expands the causal chain to include stressor -> response -> health -> vital rates -> population effect. We suggest a variety of approaches to simplify the challenging problem of estimating the combined effects of multiple stressors and to improve the estimates most relevant to management. Next, the effect of health indicators on vital rates and population status must be estimated. If the predicted effects pose too high a risk to the population, then this requires estimating what combinations of stressors can be reduced to bring the risk to an acceptable level and a plan to monitor whether the reductions are having the desired result.

Box 2. **Steps for the assessment of the cumulative risk to a wildlife population of exposure to multiple stressors:**

 1. define management goals;
 2. develop a conceptual model linking activities to stressor exposures to effects on species relevant to the management goal(s), including indirect effects through ecological interactions;
 3. develop a plan for monitoring critical indicators in the ecosystem to estimate whether the risk to the population reaches thresholds for action;
 4. use a population consequences of multiple stressors (PCOMS) modelling framework to prioritize stressor → response → effect chains of highest need for mechanistic studies involving health;
 5. develop a plan for monitoring exposure of the population to priority stressors;
 6. for each stressor prioritized for studies involving health:
  6.1. analyse the causal chain from dose → response → health → population effect‌;
  6.2. estimate the dose–response function‌; and
  6.3. use dose–response functions and distributions of stressor and populations to estimate the effect of expected dose across the population‌;
  ‌
 7. analyse risk of aggregate exposure to each individual priority stressor;
 8. accumulate the responses from all the priority stressors to estimate the combined effects on health and compare predictions to measured health indicators;
 9. estimate how health status affects vital rates and population status;
10. are the predicted combined effects or the observed ecosystem effects consistent with the management goals/thresholds?
11. if mitigation action is required, estimate what reductions in stressors could meet the goal/threshold;
12. reassess the best balance between experiments to understand the mechanisms by which each stressor causes its effect and monitoring how ecosystems respond to changes in multiple stressors; and
13. monitor progress to goal and whether doses and effects are reduced as expected.

**Figure 3.**
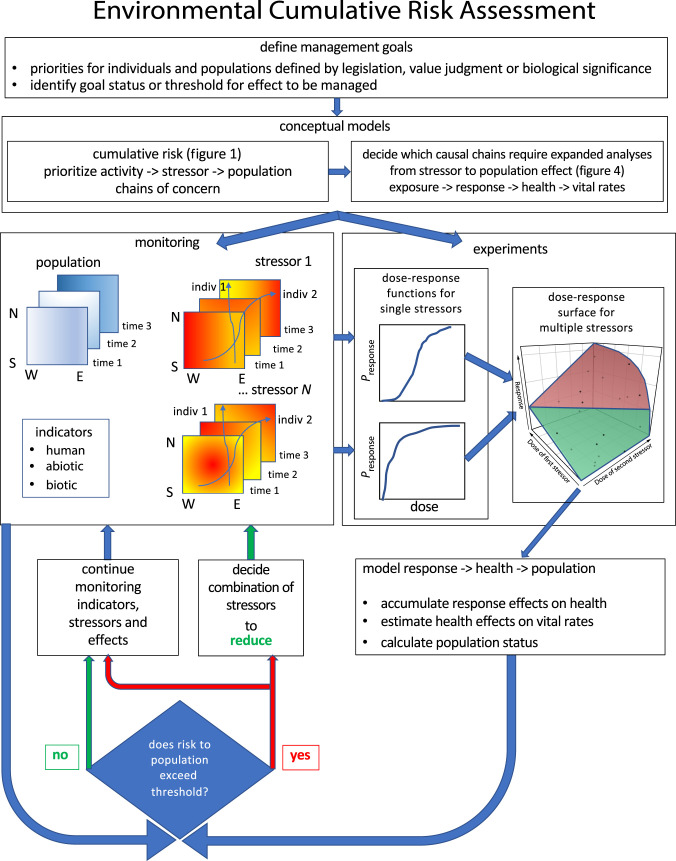
Diagram of steps suggested for integrating assessment of cumulative risk from multiple stressors into ecosystem-based management. (Online version in colour.)

The initial and final steps of our approach parallel those used in ecosystem-based management. Intermediate steps of ecosystem management often assess risk using ecosystem models or qualitative network modelling, with the outcomes estimated by monitoring ecosystem indicators [16]. These methods are not well suited to estimating the combined effects of multiple stressors, nor have they proved competent to prescribe what combinations of stressor reductions will bring a population back to the desired state [22]. Our framework can improve ecosystem-based management of these problems. The next sections of this paper will discuss each step in the approach described in box 2 and illustrated in figure 3.

### (a) Define management goals

Most cumulative effects analyses are triggered either by a proposed action, by the release of a stressor into an ecosystem, or by detection of adverse effects. Different regulations are relevant for each trigger; these typically involve specific jurisdictions and frame how to define the management goals. Some regulations require more robust estimates of cumulative risk from interactions between multiple stressors than practised in the past. Here, we focus on goals to maintain wildlife populations in a healthy state within their ecosystems. Managers should work with experts in the available science along with stakeholders to define the specific management goals that must be met; it is often useful at this stage to define threshold(s) that require management action.

### (b) Develop a conceptual model linking activities, stressors and effects

Cumulative risk and ecosystem-based management approaches typically start with a scoping process that generates a conceptual model or diagram linking activities, stressors and effects (see figure 1*b* for a simple example). The conceptual model should help to prioritize which stressors are most relevant and which species are most affected by them. This prioritization should jointly consider how stressors are generated by the activities, how exposed each species is to each stressor, which species are most sensitive to each stressor and combination of stressors, and how the different species are ecologically linked. If the assessment targets one managed wildlife population, then the model should assess all stressor pathways and ecological links that affect it.

At this stage of analysis, it is important to identify and include stakeholders, their interests and goals, and what activities and effects on resources they view as important. Discussion involving stakeholders, managers and scientists helps to identify critical activities, stressors and effects, to identify how components may interact, and to weigh costs and benefits across human and natural components. In considering the level of resources to devote to the problem, it helps to know the costs that stakeholders perceive for potential loss of resources and for different mitigation policies. Success of the process is more likely with inclusion of stakeholders who value the resources and whose activities may be affected by management actions [18]. The higher the risk to the population and the higher the cost of mitigation, the more resources should be devoted to more precise estimation of the risk and to the collection of information needed to select the best mitigation actions if the risk is too high.

### (c) Develop a plan for monitoring critical indicators in the ecosystem to estimate whether the risk to the population reaches thresholds for action

Once a conceptual model has been developed, traditional ecosystem-based management typically focuses on identifying indicators for risk to critical ecosystem resources. The abundance and growth rate of target population(s) is a common indicator. However, repeated surveys can involve long time lags between when a problem develops and when it is detected, and an observed decline seldom provides guidance as to what measures may improve the population status. The US National Academies of Sciences, Engineering and Medicine (NASEM) [8] advocates developing indicators that act as early warning signs of declining status of populations, such as increases in mortality or decreases in reproduction [26]. Monitoring health indicators for populations at risk can help identify stressors whose reduction may mitigate adverse effects on vital rates. The scarcity of indicators that are suitable for use with wildlife populations and their abundance in human medicine indicates that this is a promising area for innovative research and development.

### (d) Use a Population Consequences Of Multiple Stressors modelling framework to prioritize stressor → response → health → population effect chains of highest need for mechanistic studies involving health

Some stressors have such immediate consequences for individuals in a population that ecosystem-based managers can simply monitor the stressors and population status to identify appropriate management strategies when a target population requires action to improve its status. Many conservation efforts focus on these severe *immediate effects*, but as CEQ [6, p. 1] emphasizes ‘the most devastating environmental effects may result not from the direct effects of a particular action, but from the combination of individually minor effects of multiple actions over time’. This view emphasizes the importance of *protracted effects*, defined as ‘A stressor effect that does not immediately result in death or reproductive failure, but persists or is irreversible, and may influence long-term survival or reproductive success’ (called ‘chronic effects’ in [8, p. 133]).

From the point of view of conserving populations, the critical effects on individuals are those that affect survival or reproduction. Analysis of protracted effects is complicated by the large gap between documenting diverse short-term responses to multiple stressors and predicting the long-term consequences for the probability of survival or reproduction. We argue that the ability to predict population effects may be improved by expanding the normal stressor -> population effect model to include shorter-term functions linking stressor to *response* and response to *health*.

**Figure 4.**
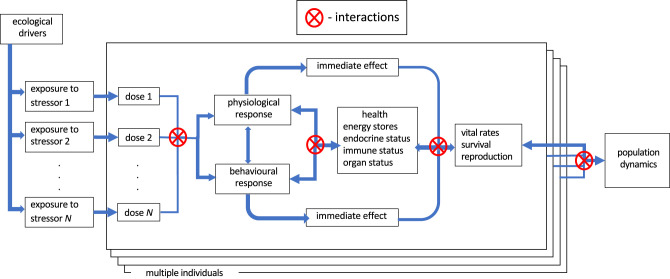
Population Consequences Of Multiple Stressors (PCOMS) conceptual model (modified from [8]). The doses caused by exposure to stressors trigger responses that can either have an immediate effect on vital rates or can accumulate changes in health which affects vital rates. The effects of stressors on population dynamics are modelled by summing these effects across all individuals. Areas where interactions between stressors, responses and population effects can occur are indicated by red circles with an ‘X’ inside (Online version in colour.)

A report from NASEM developed a Population Consequences Of Multiple Stressors (PCOMS) conceptual model that can ‘be used to understand how specific stressors affect individual animals, how these effects can accumulate as a result of exposure to multiple stressors, and how these cumulative effects may translate into population-level consequences' NASEM [8, p. 61]. A modified version of the PCOMS conceptual model is outlined in figure 4. The left-hand side shows the exposure of an individual to multiple stressors indicated by the boxes to the left of the stacked large boxes that represent individuals. *Exposure* is indicated outside of the individual, following our definition in box 2 as measured in the environment near an organism. *Dose* by contrast is measured on the animal, in a specific tissue or target organ or near the animal (for example in its prey) and is quantitative. A dose causes a response after it triggers a receptor in the individual.

The red circle with an X inside between the dose and response boxes in figure 4 indicates that different stressors can interact as they cause responses. The way to estimate the combined effect of multiple stressors depends upon the mechanisms by which they exert their effect. The joint effect of toxicants that cause the same effect by acting on the same receptor is often estimated by adding the doses of the toxicants, with a correction factor for differing potency [27]. Most stressors however require separate dose–response functions, even when they cause the same response. Challenges and methodological approaches to estimating the response caused by a combination of stressors are discussed by Boyd *et al*. [28] and Pirotta *et al*. [22].

Understanding the cumulative effects of stressors on vital rates of a population requires study of long timescales compared to the timing over which stressor exposure may vary and over which most responses persist. In some cases, it is possible to study the effect of exposure to stressors on survival or reproduction directly. If the responses caused by a stressor affect survival or reproduction immediately, these immediate effects are represented by the arrows drawn directly from the response boxes to the vital rates box in figure 4. For example, if a stressor triggers a physiological response that causes infertility or causes an animal to die, then these immediate effects on survival and reproduction would be tallied in the vital rates box.

The PCOMS framework can help prioritize which stressors require expanded analysis of stressor->response->health->population effect chains. When estimating the functions relating stressor exposure directly to population effects is problematic, NASEM [8] recommends using health indicators with an intermediate timescale between stressor exposure and immediate responses on the left side of the individual box in figure 4, and vital rates on the right side. In figure 4, protracted effects are accumulated in a ‘health box’, which integrates multiple responses that affect different indicators of health, such as energy stores, and the status of endocrine, immune and organ systems. Understanding the mechanisms by which each stressor changes health, or a health condition affects vital rates, is important to identify which stressors and health indicators are most important, how they may interact and to estimate the effects of reducing stressors [27]. Multifactorial methods that relate exposures to more than one stressor to relevant health conditions and different health indicators to survival and reproduction are well-established for human health and should be adaptable to other species.

The red circle with an X in the centre between the health and vital rates boxes in figure 4 indicates that different health indicators can interact as they affect vital rates, and also that health can modulate the extent of immediate effects on vital rates. For an example that involves interactions at several stages of the PCOMS framework, an animal in poor health may not be able to produce as strong an avoidance response to the stressor of an approaching predator, increasing the probability of being killed.

### (e) Develop a plan for monitoring aggregate exposure of the population to priority stressors

Our cumulative risk approach emphasizes monitoring exposure to stressors as well as monitoring ecosystem indicators. An animal is only exposed to a stressor that occurs in its environment, and therefore exposure assessment involves comparing the distributions of stressors to the distribution of animals. Exposure assessments require careful attention to the spatial and temporal scales of sampling based upon the distribution and biology of target populations and the stability of the stressors and their distribution. Assessment of aggregate exposure requires a careful inventory of the various potential sources of the stressor and the routes it may take to arrive at the target population (e.g. [29] for assessing noise exposure). The presence of predators, competitors, and pathogens are stressors for the target population, as is limitation of prey availability. We may fail to take the appropriate scales into account unless we consider the distribution of species that interact with the target population [30]. These other species may be affected by human activities displaced far away in space and time from where they interact with the target population (e.g. [23]).

The distinction between exposure and dose emphasizes the importance of tracing the routes and pathways from the sources through the environment to the relevant biological receptor that causes a response. For example, exposure to persistent organic pollutants in the environment around top predators may be orders of magnitude lower than the concentration in their prey, owing to biomagnification as the chemicals are transferred up the food chain [31]. In this scenario, the concentration in the prey would be a better measure for dose than concentration in the surrounding medium. If regulations are put in place to reduce exposure, it is often important to monitor changes in both exposure and dose to test whether the regulations are achieving their goal.

### (f) For each stressor prioritized for studies involving health, analyse stressor dose → response → effect chains

Stressors that expose enough of the population with potential for sufficiently intense protracted effects may be prioritized for analysis of the causal chain from the dose of the stressor to a response that affects health and from changes in health status to effects on survival or reproduction. Analysis of the mechanism(s) by which the stressor causes the response may inform the process for converting estimates of exposure to dosage of the stressor. Once dosage can be estimated, the next step is fundamental for toxicology and pharmacology but has received short shrift in studies of cumulative effects of most stressors on wildlife populations. This involves estimating the function that relates dosage of the stressor to probability and/or intensity of the relevant response. Monitoring indicators for stressors and responses in the wild can help to estimate these functions, but well-designed experimental studies can prove that the stressor causes the response. If the stressor is a good candidate for reduction to mitigate effects, and if this reduction will be costly, then demonstration of causation and how much reduction of the stressor is required to lower the response to acceptable levels may be well worth the additional cost of dose–response experiments.

Careful planning can optimize the design of dose–response experiments. Understanding the mechanism by which stressors cause the response can inform selection of functions used to estimate responses [27], and the more confident one is about the functional form, the fewer data points required to estimate the function [22]. Efficient experimental designs can be borrowed from clinical trials that estimate dose–response functions with small sample sizes. Laboratory experiments with model species may be suitable to estimate dose–response relationships that are conserved across taxa and that do not depend on context. The more taxon- and context-dependent responses are, the more important it is to conduct experiments with the relevant species in relevant contexts in its ecosystem. The need to estimate dose–response functions in wild animals or in predicted future environmental conditions has initiated the blossoming of creative methods to estimate functions such as the avoidance response of cryptic beaked whales to exposure to naval sonar at sea [32] or the effects of ocean acidification on mollusc larvae [33]. It also may be necessary to apply dose–response functions from well-studied stressors to novel, emerging or poorly studied stressors that are closely related. For example, dose–response functions derived from well-studied toxicants or sounds have been used to estimate effects of new stressors with similar chemical or acoustic properties.

### (g) Analyse risk of aggregate exposure to each priority stressor

Once a dose–response function has been estimated, the total effect on the population can be estimated by analysing the distribution of the stressor among the population. This requires estimates of stressor exposure and dosage as discussed in §6e. The more that is known about the sources of the stressor and the better our understanding of how it spreads, the less measurement is required to map stressor exposure. Estimating risk requires careful consideration about how to convert aggregate exposure to dose and how the time series of dosages drives the overall effect. Understanding the mechanism of action of the stressor is critical for estimating how to accumulate effects because the prolonged effects of repeated exposure to a single stressor accumulate in different ways depending upon the mechanisms by which the responses affect health. For example, repeated doses of a stable toxicant may accumulate leading to a response based upon the total exposure [27], but the effect on health of repeated exposures to a pathogen may decrease based upon enhanced immune responses after initial exposure. When an animal is exposed to an unstable toxicant or intense sound, the dose can be accumulated, but then decremented as the toxicant degrades or during periods of quiet when the auditory system can recover [34]. Where possible, it is helpful for estimates of the accumulated doses and effects of some stressors to be tested by measuring indicators in study populations.

### (h) Accumulate the responses from all the priority stressors to estimate the combined effects on health and compare predictions to measured health indicators

Estimating the combined effects of multiple stressors is the most challenging problem for predicting cumulative risk. Here it is critical to select the priority stressors and ranges of dose that need to be studied in combination. Boyd *et al*. [28] point out a fundamental problem for multiple stressor experiments—the sample size required equals the number of stressors times the number of dose levels times the number of replications. If only 1–2 stressors are high priority, careful selection of relevant doses can lead to tractable experimental designs that estimate how the response varies as a function of the dose of both stressors. If many stressors are high priority, analysis of their combined effects can be simplified depending on the management problem. If multiple stressors tend to occur in stable combinations, then the effects of variation in the dose of that specific combination (or a gradient towards an expected future combination) can be tested directly. Established examples include testing the toxicity to aquatic organisms of a standard set of chemicals or the combination of toxicants present in samples of effluent from a site of concern [35]. If managers propose to reduce a small set of 1–2 stressors to achieve a management goal, then the effects of proposed combinations of reduced doses can be tested against the background doses of stressors that will not be changed.

Dose–response studies are most easily studied using short-term responses that can be assessed more rapidly than changes in health. However, if these effects are shorter term than the health indicators, it is necessary to define how the shorter term effects accumulate to lead to the value of the health indicator. Energy stores are a good example of a health indicator for which theory and data allow us to link the energetic consequences of short-term responses to different stressors in terms of energy gained from foraging or spent in metabolic costs. The shared currency of calories allows energetic effects from different stressors to be integrated and accumulated in terms of effects on energy stores. The modelled longer-term consequences on energy stores can be tested by measuring energy stores directly when animals are handled, or indirectly in the field using a variety of techniques (e.g. [36]). Monitoring of these predicted health indicators is an important part of our approach, both to provide early indicators of changes in status and to test our predictions.

The health box in figure 4 contains several different health variables: energy stores, endocrine, immune and organ status. Each cumulative risk assessment should select health variables that can accumulate significant prolonged adverse effects of high-priority stressors. For example, a variety of stressors such as threats from predators or disturbance and poor energetic status can stimulate physiological systems involving the autonomic nervous system fight-or-flight response and the endocrine system's release of hormones such as corticosteroids or thyroid hormones. Monitoring of these physiological stress response systems can help estimate some of the cumulative effects of multiple stressors on health [37]. The immune system can provide markers for past and present exposure to parasite and pathogenic stressors. Monitoring these health metrics in samples of the population improves PCOMSmodelling and provides early indicators of cumulative risk. Sophisticated biomedical indicators have been developed for these metrics, but some need to be adapted for use with wildlife. For example, many traditional measures of organ function require handling the subject, which may not be practical for many wildlife species.

The potential for bidirectional interactions between responses and health must also be accounted for, as indicated by the two-way arrows and the circle with an X in the middle of figure 4. The probability or strength of an animal's response to a stressor may depend upon its current health status. For example, when disturbed, animals with low energy reserves may respond differently from healthier animals [38]. Understanding the mechanisms by which the status of one health indicator affects the response to stressors that affect other health indicators is important for estimating how they may interact. For an example of sequencing of stressors, lipophilic toxicants may be sequestered in fatty tissues and then released to receptors long after exposure when these tissues are mobilized for energy owing to the stressor of food limitation [39]. Careful study of the pathways by which stressors interact with their targets and how responses interact mechanistically to cause adverse effects at all levels of biological organization can help identify time courses and interactions that would otherwise be difficult to predict.

### (i) Estimate how health status affects vital rates and population status

Different indicators of health affect vital rates in different ways, and knowledge of the mechanisms facilitates estimating these effects. Many different disciplines recognize that energy stores and metabolism link different health states in terms of their combined effects on survival and reproduction (e.g. ecology: [40] life-history studies [41]; stress: [42]). The increased energetic cost of reproduction means that animals with low energy stores may have fewer successful offspring. For example, New *et al*. [36] modelled the relationship between a female seal's mass, the mass of her pup at weaning and pup survival, to estimate how disturbance of foraging results in lower pup survival.

Clinicians often use information integrated from a variety of health indicators to develop health scores to estimate a patient's prognosis. Veterinary science may provide some guidance for developing health scores to estimate effects on survival and reproduction for wildlife (e.g. [43]). Careful attention to mechanisms of how health indicators interact can help guide efforts to estimate survival and reproduction based upon a suite of health indicators. For example, stress hormones may suppress energetically costly immune responses especially during reproduction, which may trade off the probability of health and future survival against current reproduction [44].

Once changes in vital rates have been estimated, theory and methods are well established to predict how changes in vital rates will affect population dynamics [8]. However, interactions between individuals within the population may also affect their vital rates. This interaction is indicated by the circle with an X in the centre on the far right of figure 4. The multiple boxes indicated in figure 4 for multiple individuals are needed to quantify the variation in exposures, effects and vital rates which are used to scale up to the population. Density-dependent effects within the population may be caused when the presence of conspecifics changes the exposure to stressors. For example, conspecifics may compete for food, increasing the stressor of prey limitation.

### (j) Are the predicted combined effects and the observed ecosystem indicators consistent with the management goals/thresholds?

Different management goals will require different indicators to trigger management actions, and the level of certainty required may vary under different regulatory regimes. Populations known to be endangered may require management action until their risk of extinction falls below an acceptable level. Management actions for species that are otherwise protected may be triggered by evidence of population decline. Management for exploited species may focus on maintaining a sustainable harvest. All these goals require information on population dynamics at the far-right side of the PCOMS model (figure 4). However, as discussed above, timely management will often require early indicators such as demographic parameters or indicators of *population health*. Information from these indicators may be combined to estimate whether the status of the population is consistent with management goals.

There will almost always be uncertainty about whether and when mitigation actions need to be taken to meet management goals. Many national and international laws require a precautionary approach under which ‘lack of full scientific uncertainty shall not be used as a reason for postponing cost-effective measures to prevent environmental degradation’ [45, p. 3, 46]. However, there is often disagreement about how much evidence is required to take action. A cumulative risk approach that quantifies uncertainty can help managers make decisions based upon the probability of adverse effects.

### (k) If mitigation action is required, estimate what reductions in stressors could meet the goal/threshold

Once it has been determined that stressors must be reduced to meet a management goal, the first step is to identify which stressors that affect the target population *can* be reduced and how long it takes for the stressor reduction to take effect. For example, when development reduces critical habitat, restoration often takes decades, if it is even possible [47]. Some stressors may not be controllable if they originate from activities or locations that are outside the jurisdiction of the managers. Stressors involved in climate change or those that take effect through the food chain may originate far away and have long timelines between reduction of emissions and when effects are reduced. Timelines for recovery are slow for stressors whose effects are prolonged for years after exposure. Short-term exposure to pollutants or pathogens that permanently damage health may make animals more vulnerable to other stressors even after the initial stressor is no longer present [43].

The best candidate stressors for reduction to meet the management goal are short-lived and have critical effects on the population that diminish soon after exposure is reduced. For example, exposure and responses to sound, light or short-lived toxicants usually stop soon after emissions stop. Many of the mechanisms that animals use to respond to stressors require additional energy. This means that actions which improve availability of prey or that otherwise improve energetic status are likely to support the ability of animals to respond to other stressors. Bioenergetic models can be used to estimate how the combined effects of different stressors are modulated by energy stores [48].

Dose–response functions for each stressor that can be reduced can be coupled with information on the effects of health on vital rates to estimate how much benefit is expected for each reduction in dose. If only 2–3 stressors are candidates for reduction, then strategic selection of combinations of doses that test critical areas for reducing the response can help reduce the number of experiments required [28]. The relevant range of doses to be tested depends upon the ranges expected in the environment down to the lowest reductions that are proposed. These experiments can select the most effective combinations of stressors to reach the conservation goal, enhancing the ability of managers to negotiate with stakeholders over which stressors to reduce by how much.

### (l) Monitor progress to goal and whether doses and effects are reduced as expected

Populations are difficult to manage within complex ecosystems, and *in situ* monitoring is required to reach and maintain management goals that involve population status. We advocate adaptive management that monitors a set of indicators operating on timescales from those of short-term changes in priority stressors and responses to medium-term changes in health, to long-term changes in populations. Structuring monitoring in this way allows managers to detect and respond to deviations from the expected effects of reducing the stressors and to adapt management strategies depending on whether it is the changes in stressors, responses, health or population indicators that were unexpected. If stressors have not changed as anticipated, then regulators may need to monitor and enforce required changes to anthropogenic stressors. If a response does not change as expected, planned reduction of relevant stressors could be altered. If health indicators or early warning population indicators do not change as expected, then the model may be inadequate. Managers must also consider whether the system and/or stressors have changed over time and this variability is not accounted for in the model. This may suggest enough problems with the model to encourage consideration of additional stressors and/or alternate model formulations. The key is to enable rapid adaptation of management strategies based upon monitoring indicators of *in situ* chains of causation.

## 7. Advantages of managing cumulative risk to populations from stressors

We have argued that development of stressor-based cumulative risk assessments may provide more robust protection for wildlife and can identify more effective and practical management strategies than those resulting from many current management regimes [4]. Our approach builds upon the strengths of ecosystem-based management and adaptive management strategies; next, we discuss advantages with respect to alternative approaches.

### (a) Compared to protecting special areas

Many conservation efforts focus on establishing sanctuaries or preserves that limit human activities such as hunting [49], and protection of endangered species often focuses on critical habitats [50]. However, today some of the greatest environmental threats stem from pervasive stressors associated with activities such as those that release climate-changing gases or chemical or acoustic pollutants from multiple dispersed sources. Protected areas cannot ban these distant activities nor block exposure to stressors that propagate freely through the atmosphere, rivers or oceans. With a changing climate, critical habitats may move from protected to unprotected areas. Protected areas are important but our approach to managing the effects of stressors can directly address threats that ostensibly ‘protected’ areas are not set up to manage and can support more dynamic management of critical habitats.

### (b) Compared to an action-based approach

The analysis of cumulative risk described here also offers ways to reduce risks to managed populations that are difficult to achieve using the action-based definition of cumulative effects. In most environmental assessments, proponents analyse how a proposed human activity will affect the environment compared to alternate actions, including a no-action alternative. In effect, this sets the pre-action *status quo* as a baseline, and once each new action takes place, this establishes a new baseline for the next proposed action. Shifting baselines are a problem for environmental management [51], and the lack of clarity about how to deal with shifting baselines weakens the ability of the EIS process to manage progressive environmental degradation. CEQ [6, p. 1] states that ‘most environmental effects can be seen as cumulative because almost all systems have already been modified, even degraded, by humans’, which suggests that cumulative effects analyses should aim to address the full history of human modifications and degradations of natural environments. Our approach studies stressors accumulated from all relevant activities over time.

The way in which EISs evaluate a proposed action in the context of all other actions also limits the solution space for reducing potential impacts. For example, suppose that existing actions A and B are known to have large impacts on an endangered population, and proposed action C has a small and uncertain impact. If the proposed action C has a much higher value to society than B, the best solution might be to reduce the impacts of action B enough that allowing action C still maintains a low enough risk of adverse impacts. This is unlikely to result from the environmental assessment process but is inherent in our approach. Regular assessments that follow our approach to estimate the cumulative risk of all activities that may affect a regional ecosystem would probably be more efficient as well as better able to balance risk reduction than individual environmental assessments for each activity.

## 8. Challenges for our proposed approach

Many laws and regulations require analysis of cumulative effects, but most current analyses fail to meet the requirement (e.g. [21]). We have argued that the ability to estimate cumulative risk of multiple stressors is essential for managing effects of human activities on wildlife populations in their ecosystems. However, successful assessment of cumulative risk is hindered by inadequate science. NASEM [8, p. 10] warns that ‘the state of the science of cumulative effects has low predictive power compared to regulatory demands to assess these effects’.

We advocate the approach listed in box 2 and illustrated in figure 3, but we acknowledge that the state of theory and data on cumulative effects of multiple stressors is so poor that there is too much uncertainty today to turn our approach into quantitative predictive models for most real-world management applications. Our current inability to predict how changes in stressors affect wildlife health and how health affects vital rates means that we have low power to predict problems for populations based upon information about stressors alone. Ecologists frequently discover substantial changes in populations that are complete surprises [52]. Advancing the science to reduce these risks will require further development of modelling approaches and of methods to collect the required data on stressors, responses, health and populations at the appropriate spatio-temporal scales.

A fundamental scientific problem that hinders our understanding of cumulative effects of multiple stressors is that exposure to multiple stressors often produces effects that cannot be predicted based upon exposure to each stressor individually. We have proposed ways to simplify the problem of combined effects, but this remains a significant challenge.

## 9. Meeting the challenges

Our approach simplifies problems for analysing cumulative effects compared to separate analyses of each proposed action. Where the management goal is improving adverse population status, it may be simpler and more important to identify stressors that *can* be reduced and to estimate combinations of reduced stressor levels that restore the population to a safe status. Estimating the impact of these reductions will often depend on understanding the mechanism by which a stressor causes a response, how the accumulating responses lead to health effects, and how these changes in health affect vital rates. Biomedical science has well-developed methods to address all of these topics, but much work remains to adapt them for wildlife.

Developing the science needed to estimate the effects of different combinations of priority stressors is a major challenge but will be essential for managing the effects of multiple stressors. We have discussed several solutions that have been developed for this problem. If 1–2 stressors are the best candidate(s) for reduction to protect a managed population that is exposed to a stable combination of other stressors, then it is possible to study the effects of reducing those stressors in the context of the other stressors combined [28]. If the stressor levels are changing, then several different suites of stressors associated with different scenarios may need to be tested, and some of these suites may not be present in today's environment.

Many empirical challenges must be addressed to enable our approach to studying cumulative effects. Knowledge of the status of managed populations is seldom sufficient. Delays in assessing population status hinder effective management, so more rapid indicators of adverse status are needed for most populations. Methods are also required to:

— estimate the exposure of managed populations to human, abiotic and biotic stressors;
— test whether and how stressors cause effects, convert exposure to dose and estimate dose–response functions;
— prioritize stressors by measuring dosage across the population and using dose–response functions to estimate risk;
— estimate how the priority stressors interact to affect health;
— measure the health of wildlife with practical indicators that can estimate adverse effects; and
— study how different components of health interact to affect survival and reproduction.

The distribution and abundance of many protected species is regularly assessed, but few censuses are precise and frequent enough to detect declines with sufficient time to activate management before the problem gets worse [53]. Regulatory agencies increasingly support ecosystem-based management, but there are few ecosystems where the distribution of relevant stressors has been mapped at spatial and temporal scales suitable for modelling effects on populations. An interactive effort is needed between modellers and data providers to develop relevant models and provide data on the distribution of populations and stressors sufficient for reliable estimates of exposure and dose [54].

Many nations have legislation to protect endangered and threatened species and their habitats and have committed to international agreements to protect species against extinction, but global changes in stressors owing to climate change and other anthropogenic drivers may require new strategies to meet these goals [55]. Cumulative effects of stressors that are growing in number and strength owing to human activities are increasing extinction rates of vertebrates [56]. The total number of known extinctions is still low enough to suggest that much biodiversity can be retained, but only if the drivers of extinction can be identified and reversed [57]. Our era of climate change, human population growth, and globally expanding industrialization requires major commitments to expanding the scientific effort required to estimate the cumulative risk posed by multiple stressors on wildlife populations in their ecosystems. If we cannot estimate how to manage the effects of stressors to sustainable levels, our institutions cannot make reasonable decisions about how to protect wildlife populations from the cumulative risk.

Our societies spend significant resources dealing with issues related to cumulative effects every year. We spend billions to react to disasters and to plan projects, but we lack a commensurate investment in the research and management infrastructure required to meet our environmental commitments and goals. In some ways, the current situation is not unlike the condition of human medicine around the start of the twentieth century. Ecosystems and human health are both stabilized by complex feedback mechanisms that make it hard to predict what combinations of stressors risk transition to an adverse state. Biomedical science advanced by focusing on understanding mechanisms that cause disease and setting high standards for evidence of causation and of outcomes of treatments, coupled with recognition of the scale of effort required to prevent the causes and reduce the risks of disease. Investment in environmental science and management has not been up to this task. Improving our capacity for cumulative risk assessment requires not only increased investment in filling critical data gaps, but also an equally important investment in basic research into the theory and methods required to estimate the cumulative risk of stressors on wildlife populations. This will require a commitment to grow institutions that can support the basic research required for risk assessment; the applied science required to monitor stressors and the health and status of wildlife populations; to estimate risks in each critical ecosystem; and to engage all stakeholders in managing these risks. Few would argue against the investments that biomedical institutions of the twentieth century made to develop science-based interventions that prolong life and enhance the health of most humans. For humans and global ecosystems to thrive in the twenty-first century and beyond, we may require the development of institutions for ecological research and management similar in scope and investment to the biomedical institutions of the twentieth.

## Data Availability

The data are provided in the electronic supplementary material [58].
